# Supplementary material for: Quantitative Detection of Mastitis Factor IL-6 in Dairy Cow Using the SERS Improved Immunofiltration Assay
Source: Nanomaterials (Basel). 2022 Mar 26;12(7):1091. doi: 10.3390/nano12071091 (PMC9000223; doi:10.3390/nano12071091)
Supplement: Supplementary file 1 [file nanomaterials-12-01091-s001.zip › nanomaterials-1650439-supplementary.pdf]

Supplementary Materials

Quantitative Detection of Mastitis Factor IL-6 in Dairy Cow Using the SERS Improved Immunofiltration Assay

Ruipeng Chen [1,*], Hui Wang [1], Yiguang Zhao [1], Xuemei Nan [1], Wensong Wei [2], Chunmei Du [1], Fan Zhang [1], Qingyao Luo [1], Liang Yang [1,*] and Benhai Xiong [1,*]

1 State Key Laboratory of Animal Nutrition, Institute of Animal Science, Chinese Academy of Agricultural Sciences, Beijing 100193, China; wanghui_lunwen@163.com (H.W.); zhaoyiguang@caas.cn (Y.Z.); xuemeinan@126.com (X.N.); duchunmeim@163.com (C.D.); zhangfan19@139.com (F.Z.); luoqingyao@caas.cn (Q.L.)

2 Institute of Food Science and Technology, Chinese Academy of Agricultural Sciences, Beijing 100193, China; weiwensong@caas.cn

* Correspondence: chenruipeng@caas.cn (R.C.); yangliang@caas.cn (L.Y.); xiongbenhai@caas.cn (B.X.); Tel.: +86-010-62811680 (B.X.)

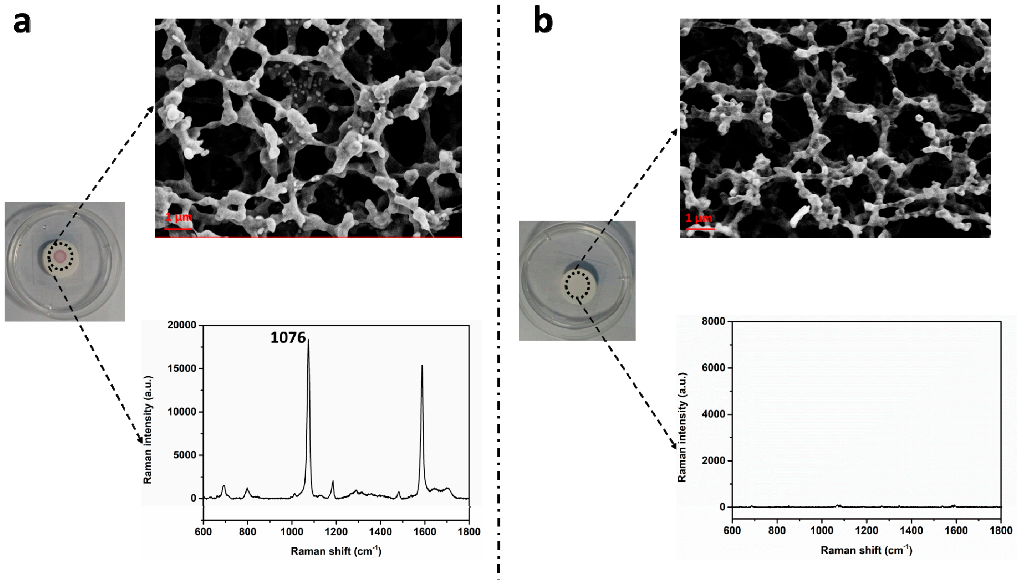

Figure S1 Feasibility test of the SERS improved IFA. The test with (**a**) and without IL-6 (**b**), and the typical pictures of their corresponding SEM images and SERS spectra of the test dots, respectively.

Table S1 Comparison of different detection methods for inflammatory biomarker IL-6.

No.	Method	Limit of detection (LOD)	Literature
1	LA	0.5 pg mL^{-1}	[1]
2	IA	1.6 pg mL^{-1}	[2]
3	CLIA	0.5 pg mL^{-1}	[3]
4	EIS	1.0 pg mL^{-1}	[4]
5	SERS	0.54 pg mL^{-1}	[5]
6	SERS nanotag	0.35 pg mL^{-1}	This work

Table S2 The detection and recoveries test of the spiked IL-6 in milk sample.

NO.	ECLIA[a] (ng mL^{-1})	SERS VFA[b] (ng mL^{-1})	Recovery (%)	RSD[c] (%)
1	0.18	0.16±0.07	88.9	8.7
2	35.7	32.8±5.76	91.9	5.2
3	167.4	145.6±13.36	87.0	4.7
4	236.5	242.8±15.08	102.7	2.5
5	504.1	487.2±12.65	96.7	6.4

[a]ECLIA is the abbreviation of electrochemiluminescent immunoassay.

[b]The average value is calculated from three repeats.

[c]RSD is short for relative standard deviation.

References

[1] Rathnayake, N.; Akerman, S.; Klinge, B.; Lundegren, N.; Jansson H.; Sorsa, Y.T.; Gustafsson, A. Salivary biomarkers for detection of systemic diseases. *Plos One*, **2013**, *8*, e61356−e61361.

[2] Tertis, M.; Leva, P.I.; Bogdan, D.; Suciu, M.; Graur, F.; Cristea, C. Impedimetric aptasensor for the label-free and selective detection of interleukin-6 for colorectal cancer screening. *Biosens. Bioelectron.* **2019**, *137*, 123–132.

[3] Luo, L.R.; Zhang, Z.J.; Hou, L.Y.; Wang, J.L.; W. Tian, The study of a chemiluminescence immunoassay using the peroxyoxalate chemiluminescent reaction and its application, *Talanta*, **2007**, *72*, 1293–1297.
[4] Barman, S.C.; Sharifuzzaman, M.; Zahed, M.A.; Park, C.; Yoon, S.H.; Zhang, S. P.; Kim, H.; Yoon, H.; Park, J.Y. A highly selective and stable cationic polyelectrolyte encapsulated black phosphorene based impedimetric immunosensor for interleukin-6 detection, *Biosens. Bioelectron.* **2021**, *186* 1873–4235.
[5] Wang, X.M.; Ma, L.; Hu, C.M.; Liu, T. W.; Sun, S. J.; Liu, X. H.; Guan, M. Simultaneous quantitative detection of IL-6 and PCT using SERS magnetic immunoassay with sandwich structure, *Nanotechnology*, **2021**, *32*, 255702.
